# Dataset: Feminine hygiene product lifecycle inventory and impact assessment

**DOI:** 10.1016/j.dib.2019.104851

**Published:** 2019-11-21

**Authors:** Amy Hait, Susan E. Powers

**Affiliations:** a Clarkson University, Department of Civil & Environmental Engineering and the Honors Program, USA; b Clarkson University, Institute for a Sustainable Environment, USA

**Keywords:** Lifecycle assessment, Sanitary products, Menstrual cup, Gabi, Dataset, Menstruation

## Abstract

A life cycle assessment (LCA) of feminine hygiene products was completed with three samples considered representative of sanitary pads, tampons and menstrual cups. Gabi LCA software was used to organize inventory data with the ILCD (v.1.0.10) life cycle impact assessment method used to determine mid-point and normalized impact scores. Data from the ecoinvent database, and literature were used to complete the assessment. The dataset includes product details (mass and materials), life cycle inventory (LCI) data and life cycle impact assessment (LCIA) results for all mass and energy flows. Hait and Powers [1] used the data in a comparative LCA.

**Table:** Specifications Table

Subject	Environmental engineering
Specific subject area	Waste Management and Disposal
Type of data	Table
How data were acquired	Life cycle assessment using Gabi software, ecoinvent inventory data and ILCD lifecycle impact assessment method
Data format	Raw and Analyzed data
Parameters for data collection	Three representative samples selected to identify materials and mass of products (sanitary pad, tampon, menstrual cup). U by Kotex® Security® Maxi Pads and U by Kotex® Click® Tampons [2]. Menstrual cup produced by DivaCup [3]
Description of data collection	The materials that comprise these products were identified primarily based on manufacturer specifications and through literature review. Both Kimberly-Clark and DivaCup list the materials in each of their feminine hygiene products on their respective websites. The average total weight and composition of tampons and sanitary pads were directly measured. GaBi LCA software [4] was used to construct life cycle plans for each of the menstrual products as well as to populate those plans with processes in the life cycle of each product. The GaBi package includes a database of life cycle inventories for the production of several materials and energy sources. ecoinvent, v3.3 [5] database was also used for materials not available directly through GaBi.
Data source location	Inventory data from the United States were selected when available; alternative inventories were used in decreasing order of preference from a global average, European average, or other country-specific analysis.
Data accessibility	Repository name: Mendeley Data Data identification number: doi:10.17632/vj6ztpt96d.1 Direct URL to data: https://data.mendeley.com/datasets/vj6ztpt96d/2
Related research article	Amy Hait, Susan E. Powers [1] The Value of Reusable Feminine Hygiene Products Evaluated by Comparative Environmental Life Cycle Assessment Resources, Conservation & Recycling DOI <to be completed when paper finalized>

**Table:** 

**Value of the Data** • LCI and LCIA data can be used to identify specific environmental and health impacts of these products • These data can be used by other researchers or by stakeholders that are interested in particular types of products to identify sources of impacts and/or benefits of the products. • These data can be used by other researchers for benchmarking and comparative purposes. There is substantial opportunity to add to this dataset with other specific products to evaluate particular material and design decisions within each type of sanitary product to reduce environmental and health impacts • There were no other datasets for feminine hygiene products readily available in the literature.

## 1 Data

**Fig. 1:**
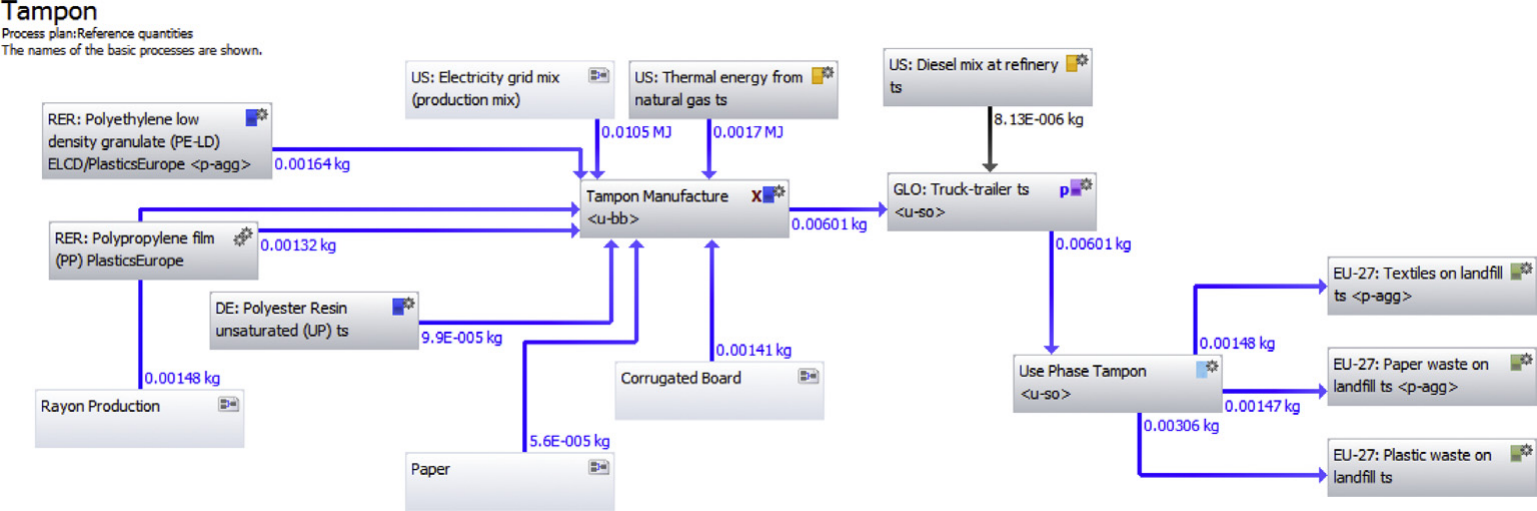
Gabi LCA software life cycle model for tampon.

**Fig. 2:**
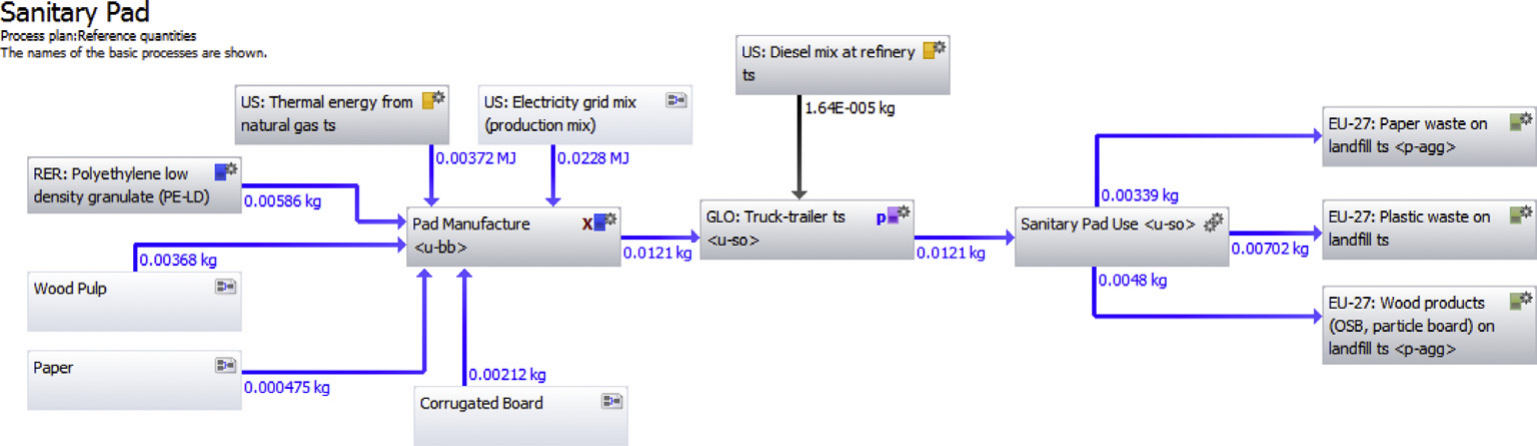
Gabi LCA software life cycle model for sanitary pad.

**Fig. 3:**
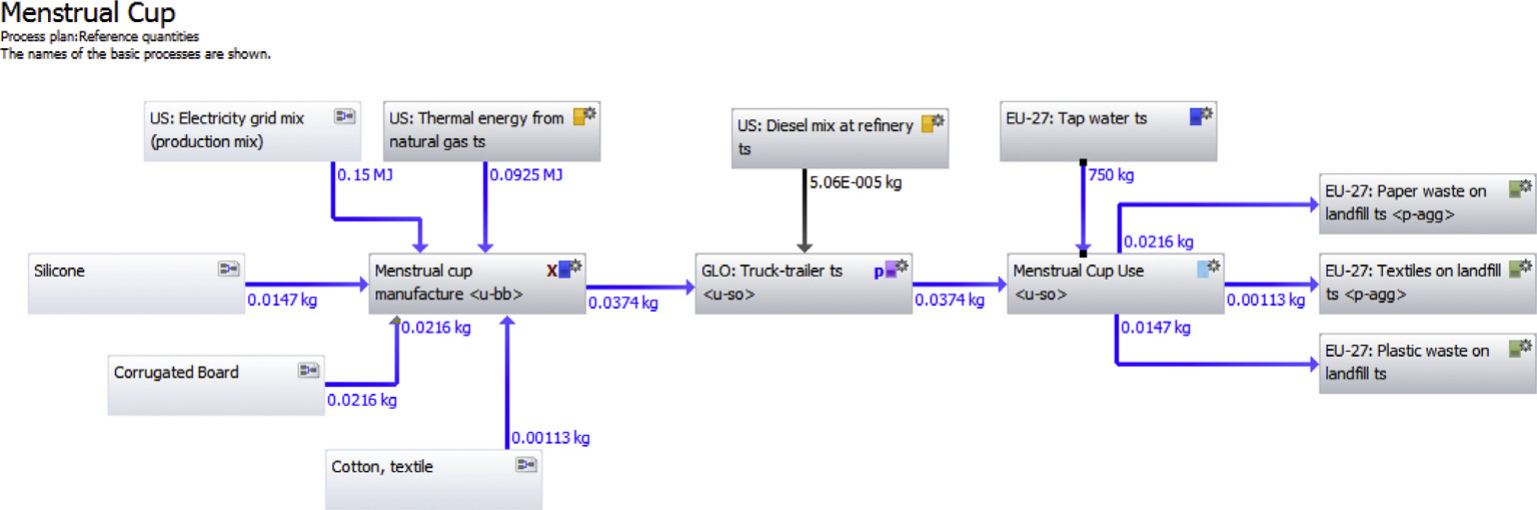
Gabi LCA software life cycle model for menstrual cup.

The dataset available through Mendeley includes input data used to build the LCA and resulting inventory and impact assessment results. The dataset is an MS Excel spreadsheet that has separate tabs for each of the three products for both LCI and LCIA steps in the LCA. Specific weights and materials of construction of each of the three products are also included in the spreadsheet. These were used to create the life cycle flows for each of the products, which are included in this DIB article (Fig. 1, Fig. 2, Fig. 3). The impact categories considered include:

• Climate change midpoint, excl biogenic carbon
• Climate change midpoint, incl biogenic carbon
• Human toxicity midpoint, cancer effects
• Human toxicity midpoint, non-cancer effects
• Ecotoxicity freshwater midpoint
• Acidification midpoint
• Eutrophication freshwater midpoint
• Resource depletion, mineral, fossils and renewables, midpoint

## 2 Experimental design, materials, and methods

Materials that comprise these sanitary products were determined from manufacturer specifications and literature resources. Key resources include: Kimberly-Clark [2]; Barman et al. [6]; SCA's 2014 Sustainability Report [7]; DOW Corning [8].

Four life cycle stages were assessed within the LCA to provide a cradle to grave analysis of each menstrual product: raw materials production, product manufacture, transportation and use, and disposal. The functional unit of the impact analysis was one year of coverage for one woman. This amounted to an average of 240 pads or tampons or 1/10th of a menstrual cup. The weight of the basic components of each sample were measured directly (average of 10 items). Other assumptions related to materials and their production are identified in the dataset. Sensitivity analyses to assess uncertainty included the number of units used per year, resources used for electricity production, the relative weight of polyethylene and cellulose in the pad's absorbent core and the inclusion of an applicator for the tampon. Gabi LCA Software was utilized to create life cycle models of the three products studied. Fig. 1, Fig. 2, Fig. 3 depict the life cycle plans of each product that were used to organize the inventory analysis and eventually the impact assessment. Each gray box in the plan represents a process in the life cycle such as raw material production, or product manufacture. The text within the box describes the process. The blue and black arrows represent material or energy flows from one process into another. The blue and black numbers besides these arrows quantify the flow from each process.

Each of the gray box processes incorporate other input and output elementary flows that are compiled by the software to create one composite inventory list with all elementary flow inputs and outputs into the system. For example, water is required in the production of both polyethylene and polypropylene. Water is not included in the life cycle diagram for readability. This elementary flow is included as part of the polyethylene or polypropylene production process. Because many processes required water, the Gabi software sums the water demand in each process and compute a total water requirement.

Some of the processes within the feminine hygiene product plans did not have inventories completed down to the elementary flow level. For such processes that were not completely defined by elementary flows, a life cycle diagram plan was created to describe that processes life cycle by elementary flows. These extra plans were then nested into the feminine hygiene product plans as individual processes. This plan nesting allows the data from the flows required in those required plans to be pulled into the feminine hygiene product plan. Thus, a comprehensive inventory was completed, without requiring every process required in one plan such as the figure below. Instead, the Gabi LCA software continues to work as if there are largely extended plans, without requiring that pictorial representation.

A search function within GaBi allows the user to view all relevant inventories and select the best suited inventory to describe the material and energy of interest. Criterion for selection of the inventory for this study was based on trying to select the best available representative inventory. If multiple options of inventories that described the material and energy flow accurately were available, preference was shown for analyses done in the United States. If US inventories were not available, alternative inventories were selected, in decreasing order of preference, from a global average, European average, or other country-specific analysis. If there were multiple methods of producing a material or energy flow, a technology mix or production mix was selected in preference to one specific method.

The inventories included in the Gabi database are not all cradle-to-grave and thus are supplemented with further material and energy process flows. Additionally, not all material and energy flows required in the production of menstrual products are included in the GaBi database. Therefore, the ecoinvent (3.3) database was used to fill in such data gaps. The most relevant study was selected by finding the LCA completed for the material that has properties most similar to that of the material used in the feminine hygiene product. Again, preference was shown for US studies and if not available, global averages. Once selected, the study provides a spreadsheet of all material and energy inputs and outputs for manufacture of the material. The data gleaned from ecoinvent was imported into feminine hygiene product inventory models in Gabi to ensure relevant material and energy processes were described as precisely as feasible.

Some inputs into the life cycle of the feminine hygiene products were excluded from the scope of this work. These materials were not easily characterized in the available database and composed a small portion of the product by weight. Based on the LCI of raw materials used to manufacture the products, only 0.76%, 0.36%, and 3.47% of the weight of tampons, sanitary pads, and menstrual cups, respectively were omitted from the analysis.

The Gabi software was also used to determine mid-point and normalized mid-point categories for each of several indications. ILCD methodology [9] for mid-point analysis was selected to use suitable characterization factors and contributing flows [10]. Mid-point scores were divided by the normalization factor to provide a single overall and unitless score for each product.

## References

[1] Hait A., Powers S.E. (2019). The value of reusable feminine hygiene products evaluated by comparative environmental life cycle assessment. Res. Conserv. Recycl..

[2] Kimberly-Clark (2014). U by kotex® Ingredients. http://www.productstewardshipatkc.com/ourbrands/kotex.https://www.kimberly-clark.com/en/brands/ingredients/consumer/kotex.

[3] Diva International Inc. (2015). Quality and standards. http://divacup.com/about-us/quality-and-standards/.

[4] Thinkstep (2016). GaBi education. https://www.thinkstep.com/software/gabi-lca.

[5] ecoinvent The ecoinvent Database (3.3). http://www.ecoinvent.org/database/database.html.

[6] Barman A., Asagekar S.D., Katkar P. An overview on sanitary napkins. https://www.technicaltextile.net/articles/an-overview-on-sanitary-napkins-7850.

[7] SCA (2014). Sustainability report. Sweden. https://www.sca.com/globalassets/sca-engelska/financial-reports/2014/sustainability-report-2014.pdf.

[8] DOW Corning (2015). The 2015 corporate sustainability report. http://www.dowcorning.com/content/publishedlit/sustainability-report-en.pdf.

[9] European Commission - Joint Research Centre - Institute for Environment and Sustainability (2010). International Reference Life Cycle Data System (ILCD) Handbook - General Guide for Life Cycle Assessment - Detailed Guidance. First Edition March 2010. EUR 24708 EN.

[10] European Commission - Joint Research Centre (2016). Developer: ILCD compliant normalization factors. https://eplca.jrc.ec.europa.eu/LCDN/developerILCD.xhtml.

